# Human microRNAs preferentially target genes with intermediate levels of expression and its formation by mammalian evolution

**DOI:** 10.1371/journal.pone.0198142

**Published:** 2018-05-24

**Authors:** Hisakazu Iwama, Kiyohito Kato, Hitomi Imachi, Koji Murao, Tsutomu Masaki

**Affiliations:** 1 Life Science Research Center, Kagawa University, Ikenobe, Miki-cho, Kita-gun, Kagawa, Japan; 2 Department of Gastroenterology and Neurology, Faculty of Medicine, Kagawa University, Ikenobe, Miki-cho, Kita-gun, Kagawa, Japan; 3 Department of Endocrinology and Metabolism, Faculty of Medicine, Kagawa University, Ikenobe, Miki-cho, Kita-gun, Kagawa, Japan; Laboratoire de Biologie du Développement de Villefranche-sur-Mer, FRANCE

## Abstract

MicroRNAs (miRNAs) are short, endogenous RNAs that post-transcriptionally repress mRNAs. Over the course of evolution, many new miRNAs are known to have emerged and added to the existing miRNA repertoires of drosophilids and vertebrates. Despite the large number of miRNAs in existence, the complementary pairing of only ~7 bases between miRNAs and mRNAs is sufficient to induce repression. Thus, miRNA targeting is so widespread that genes coexpressed with a miRNA have evolved to avoid sites that are targeted by the miRNA. Besides this avoidance, little is known about the preferential modes of miRNA targeting. Therefore, to elucidate miRNA targeting preference and avoidance, we evaluated the bias of the number of miRNA targeting occurrences in relation to expression intensities of miRNAs and their coexpressed target mRNAs by surveying transcriptome data from human organs. We found that miRNAs preferentially target genes with intermediate levels of expression, while avoiding highly expressed ones, and that older miRNAs have greater targeting specificity, suggesting that specificity increases during the course of evolution.

## Introduction

MicroRNAs (miRNAs) are ~22-nt endogenous RNAs that modulate mRNAs post-transcriptionally [1–3]. During the course of evolution, miRNAs are documented to have undergone rapid birth-and-death turnovers for drosophilids [4], vertebrates [5], and mammals [6,7]. As a result, many newly-emerged miRNAs have been added to the existing miRNA repertoire. In humans, this was estimated to consist of ~1500 loci annotated in the Ensembl Genes 89 genome assembly [8] or ~1800 in the miRBase 21 database [9], with a lower limit of ~500 loci in the MirGeneDB database, which adopts strict criteria for including miRNAs [10].

The complementary pairing of only six or seven bases of seed sequences at 5′ positions 2–7 or 2–8 of a mature miRNA with target mRNAs is sufficient to induce repression in animals [1–3]. Therefore, targeting by miRNAs is so widespread that newly-emerged miRNAs likely perturb the established regulatory networks. Such young miRNAs tend to be expressed at low levels [7,11] and are tolerated by the gene regulatory networks; of these, only a few survive and gradually grow in expression intensity [4] Genes preferentially co-expressed with a miRNA have evolved to selectively avoid sites that are targeted by the miRNA, which is known as selective avoidance [12,13]. As an extreme manifestation of this avoidance, ubiquitously expressed genes tend to have shorter 3′ untranslated regions (UTRs) [14].

These findings explain the mechanism of miRNA targeting avoidance; however, the conditions whereby miRNA targeting is preferentially accepted are largely unknown. Because many new miRNAs emerge, increase in expression intensity, and are involved in a diverse range of functions [12], we regard the miRNA targeting specificity from the relationship of the expression intensities between miRNAs and mRNAs, rather than as the function of the targeted mRNAs. In this study, therefore, we aimed to elucidate: (i) how the opposite directions of avoidance and preference of miRNA targeting are resolved into an integrated targeting specificity, and (ii) whether this specificity is shaped through evolution. To do this, we examined mRNA and miRNA transcriptome datasets from human organs.

## Results

### Lower expression of younger miRNAs with exceptions in the thyroid, testis, and placenta

**Fig 1:**
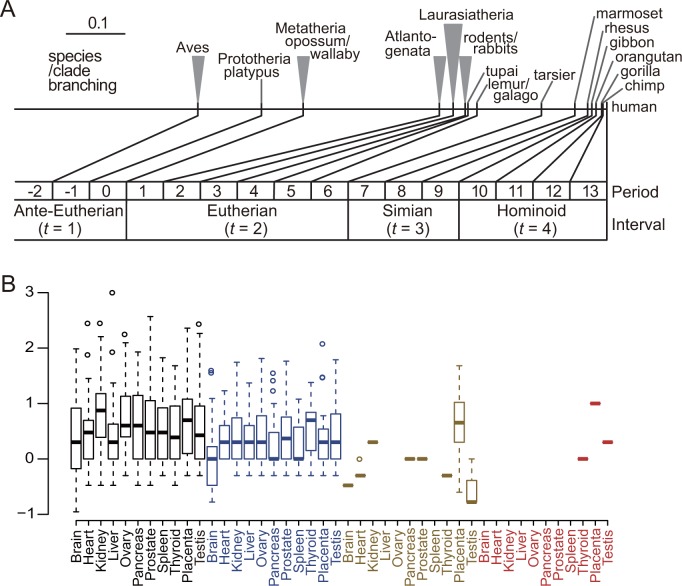
Outline of miRNA age and expression by age and by organ. (A) Divergence of the mammalian species/clades and the assignment of intervals of human miRNA times of origin representing the miRNA age. The intervals *t*s are 1: ante-eutherian, 2: eutherian, 3: simian, and 4: hominoid. The top horizontal line is proportional to the neutral distance [6]: the scale is shown in the upper left with a bar of 0.1 neutral substitutions per site. (B) Expression intensities of human miRNAs by organ and by miRNA age. The RNA-seq read counts of miRNAs in each human organ reported by Landgraf et al. [15] are plotted as the base-10 logarithm against the different evolutionary ages of miRNAs: ante-eutherian (black), eutherian (blue), simian (gold), and hominoid (red).

In this study, the age of each of 1426 human miRNAs, which was represented by the time of origin [6], was assigned to one of four time intervals: (i) ante-eutherian, (ii) eutherian, (iii) simian, and (iv) hominoid; the boundaries between these were set as the time of divergence of metatherian mammals, tarsiers, and gibbons, respectively (Fig 1A). We used an RNA sequencing (RNA-seq) dataset of human miRNAs [15] for 10 organs. We also obtained another RNA-seq dataset of mRNAs [16] for 18,951 human protein-coding genes as potential miRNA targets. To examine the trend of miRNA expression intensity of different ages, we arranged the miRNAs by age and by organ and plotted their expression intensities. Consistent with previous reports [7,11,17], older miRNAs showed higher expression intensities as a whole (*P* = 2 × 10^−4^, Jonckheere–Terpstra trend test), although a small number of simian and hominoid miRNAs showed exceptionally high expression levels in the thyroid, placenta, and testis (Fig 1B).

Simian- and hominoid-origin miRNAs were expressed at such low levels that they included few miRNAs coexpressed substantially with the target gene. Consequently, we compared ante-eutherian and eutherian miRNAs in the following analyses.

### Characteristics of human miRNA targeting specificity

We quantified miRNA targeting preference and avoidance using the bias that indicates how much the observed number of miRNA targeting occurrences deviates from the expected number. This bias is given as a function of four factors: (i) the expression intensity of the miRNA *j* (ranks 1–5), (ii) that of the target mRNA *i* (ranks 1–5), (iii) the age of the miRNA *t* (intervals 1–4;) (Fig 1), and (iv) the organ *z* (organs 1–10) in which miRNA and mRNA are coexpressed. The ranks were arranged in increasing order of expression intensity. In particular, rank 1 included every mute miRNA or gene, (i.e., with no expression in the organ of interest but with detectable expression in at least one other organ). We regarded the presence of predicted miRNA target sites as a proxy of the occurrence of miRNA targeting, as long as the miRNAs and the target genes were coexpressed in an organ.

**Fig 2:**
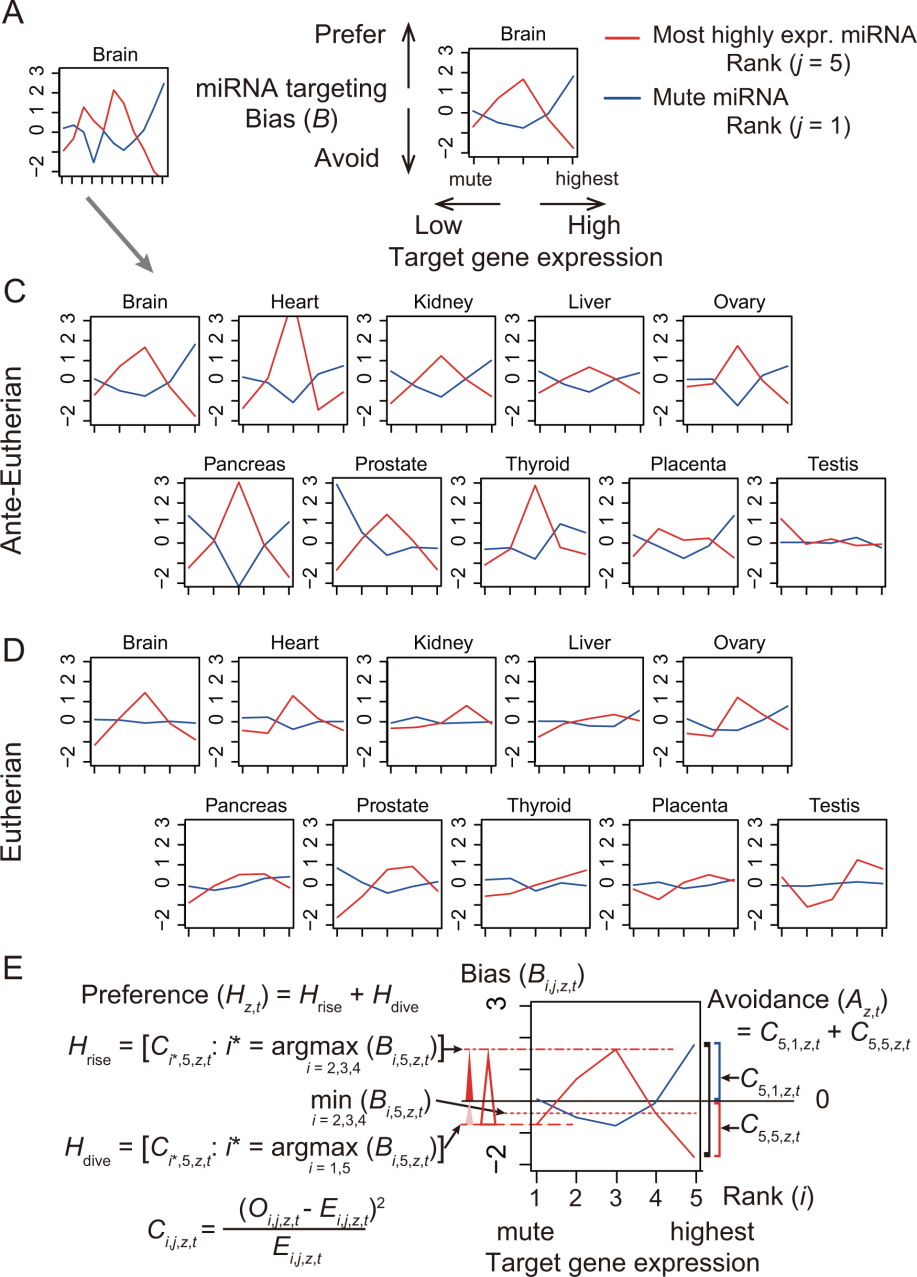
miRNA targeting specificity. The bias reflecting how the observed number of miRNA targeting occurrences deviates from the expected number is plotted against the expression ranks of target genes for the most highly expressed (red) and mute (blue) miRNAs in each organ. The bias is indicated using Pearson’s chi-squared test statistic with its log_10_ p value being signed plus when larger than expected and minus when less. (A) When the intensity of gene expression is graded using 11 levels (I–XI), the graph is extremely jagged so the expression intensity of the genes was converted into five ranks of grading to smooth the plots. This example plot is for the case where the brain is the organ and the miRNA time of origin is ante-eutherian. (B) An explanation of the plot based on the five-rank grading of the expression intensity of the genes. (C) Plots of the targeting bias of miRNAs of ante-eutherian origin for the 10 organs. (D) Plots of the targeting bias of miRNAs of eutherian origin. (E) Quantitative measures of the avoidance index *A*_*r*,*t*_ and the preference index *H*_*r*,*t*_ are illustrated. *A*_*r*,*t*_ indicates the extent of the targeting avoidance of the most highly expressed miRNAs to the most highly expressed target genes, and *H*_*r*,*t*_ indicates the extent of the targeting preference of the most highly expressed miRNAs to genes with intermediate expression for organ *z*, and for miRNA age *t*. *A*_*r*,*t*_ corresponds to the distance between blue and red lines at the right-hand side of the plot, while *H*_*r*,*t*_ corresponds to the height of the red line in the intermediate ranges (ranks 2–4) of gene expression intensity. A red open triangle corresponds to *H*_*r*,*t*_, which is the sum of *H*_rise_ (closed red triangle) and *H*_dive_ (closed pink triangle); a black bracket corresponds to *A*_*r*,*t*_, which is the sum of *C*_5,1,z,*t*_ (blue bracket) and *C*_5,5,z,*t*_ (red bracket) (see Materials and Methods).

We computed the bias *B* to measure how often miRNA targeting occurred compared with the expected frequency; the bias was indicated as positive when targeting occurred more often than expected, and negative when it occurred less often (see details in Materials and Methods). We focused on the bias of the most highly expressed (rank 5) miRNAs *B*_*i*,5,*z*,*t*_ and, in contrast, that of the mute (rank 1) miRNAs *B*_*i*,1,*z*,*t*_, and plotted them against the expression rank of the target genes (Fig 2).

First, for the old conserved ante-eutherian miRNAs, we plotted the bias *B′* (11-level grading of gene expression intensity) (Figs 2A and S1); the biases of the most highly expressed and mute miRNAs fluctuated markedly in opposite directions. Because the bias *B′* (11-level grading) plots were extremely jagged, we smoothed them based on the bias *B* (five-rank grading) (Fig 2B and 2C). Among all organs examined, except for the testis (Fig 2C), the most highly expressed miRNAs showed a Λ-shaped curve in each plot, suggesting that they targeted genes with intermediate levels of expression (i.e., ranks 2–4) more often than expected.

In the plot (Fig 2C), the red line makes a sharp descent on the right-hand side, which indicates that the number of occurrences of the most highly expressed miRNAs targeting the most highly expressed genes was significantly less than expected; in contrast, the blue line makes a sharp ascent, suggesting that the mute miRNAs had a far greater number of target sites than expected for the most highly expressed genes (which themselves have no effect because the miRNAs are unexpressed). These results demonstrated that the old conserved human miRNAs preferentially target genes of intermediate expression while avoiding targeting the most highly expressed genes that were coexpressed with the miRNAs.

### The formation of miRNA targeting specificity over the course of evolution

To elucidate whether the targeting specificity is intrinsic to miRNAs or arose during the course of evolution, we compared specificity between ante-eutherian and eutherian miRNAs. The plots of eutherian miRNAs (Fig 2D) showed that the overall trends of targeting specificity, i.e., the Λ shape formed by the sharp descent at the end of the plots, were similar to those of the old ante-eutherian miRNAs, except in the testis (Fig 2C). However, the amplitude of fluctuation tended to be smaller for eutherian miRNAs.

To quantitatively assess the extent of targeting specificity, we devised two indices and one criterion (Fig 2E, see Materials and Methods): (i) the avoidance index *A*_*z*,*t*_, which quantifies the sharp descent on the right-hand side of the plot, i.e., the extent of avoidance of targeting the most highly expressed genes by the most highly expressed miRNAs; (ii) the preference index *H*_*z*,*t*_, which quantifies the height of Λ, i.e., the extent of preference for genes with intermediate expression in organ *z* and at the age of miRNA *t*, and (iii) the Λ criterion, which defines the upward convexity of the Λ shape.

The assessments showed that both the avoidance index *A* and the preference index *H* were significantly larger for ante-eutherian than for eutherian miRNAs across the organs of interest (Table 1) (*P* = 0.004 and *P* = 0.01, respectively; signed-rank test). Additionally, the Λ shape was visible in the plots of more organs (8/10) for ante-eutherian miRNAs than for eutherian ones (4/10).

**Table 1:** Quantification of miRNA targeting specificity.

Indicator	Origin	Br	He	Ki	Li	Ov	Pa	Pr	Th	Pl	Te	p*
Avoid. Index *A*	Ante	35	11	7.7	9.1	7.0	4.0	4.0	3.6	6.2	-	0.004
	Euth	-	2.5	-	4.6	-	0.8	0.8	0.3	-	-	
Prefer. Shape	Ante	-	Λ	Λ	Λ	Λ	Λ	Λ	Λ	Λ	-	-
	Euth	Λ	Λ	-	Λ	-	Λ	-	-	-	-	
Prefer. Index *H*	Ante	-	12	1.6	5.7	2.1	5.0	2.6	2.1	4.1	-	0.01
	Euth	1.0	5.2	-	3.9	-	1.8	1.9	0.7	-	-	

Ante and Euth represent ante-eutherian and eutherian, respectively. Λ represents the bias of the most highly expressed miRNAs showing upward convexity in the plot that meets the criterion of the Λ shape; this indicates that miRNAs preferentially target genes with intermediate expression levels. Hyphens indicate non-Λ-shaped or not available. Br, Brain; He, Heart; Ki, Kidney; Li, Liver; Ov, Ovary; Pa, Pancreas; Pr, Prostate; Th, Thyroid; Pl, Placenta; Te, Testis.

*Derived using Wilcoxon signed-rank test (two-sided).

These results demonstrated that the avoidance of targeting highly expressed genes by miRNAs and their preference for genes with intermediate expression are more pronounced for old conserved miRNAs than for younger ones. This supports the notion that miRNA targeting specificity has formed over the course of evolution.

### Robustness toward different settings of miRNA target prediction

Because we regarded predicted miRNA target sites in coexpressed genes as miRNA targeting occurrences, it was pivotal for us to confirm that our findings are robust against perturbations of target prediction methods and their parameters. We first performed TargetScan analysis [18] with context++ score (CS) < −0.1, −0.2 and −0.3; each set of predicted target sites was named C010, C020, and C030, which had 0.07, 0.05, and 0.03 predicted target sites per (mature) miRNA per gene, respectively (note that our main results were based on C020.) We next used the PITA algorithm [19] to yield predicted target sites closest in number to each of the C0X0s; this resulted in PITA thresholds of *ΔΔG* < 0.96, −3.69, and −-9.49, yielding C0X0-equivalent sets named P010, P020, and P030, respectively.

**Fig 3:**
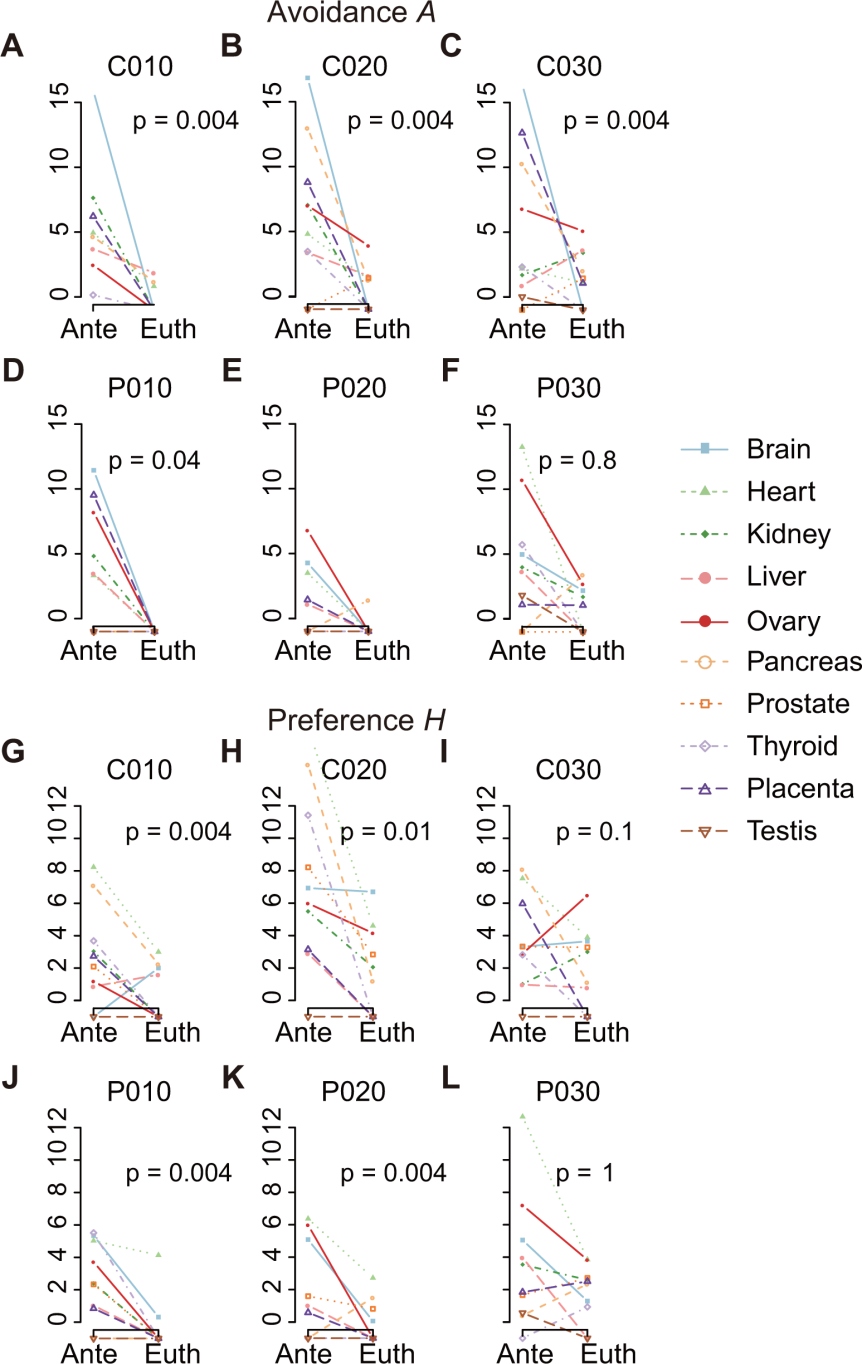
Results are robust against different methods, TargetScan and PITA, with varied stringencies. Ante and Euth stand for ante-eutherian and eutherian origins of miRNAs, respectively. (A–C) Based on TargetScan-predicted sets of miRNA target sites with an increasing level of stringency, named C010, C020, and C030, the extent of miRNA targeting avoidance (avoidance index *A*) is compared between Ante (old) and Euth (younger) miRNAs. (D–F) Based on PITA-predicted sets of target sites with an increasing level of stringency, named P010, P020, and P030, the avoidance index *A* is compared between Ante and Euth miRNAs. The avoidance index of Ante is greater than that of Euth for almost all of the 10 human organs examined and over the various levels of stringency, except for (F) the PITA most stringent set P030. (G–I) TargetScan-predicted sets of C010, C020, and C030 are applied to compute preference index *H* and compare *H* between Ante and Euth miRNAs. (J–L) PITA-predicted sets of P010, P020, and P030 are applied to compare the preference index *H* between Ante and Euth miRNAs. For every plot, when the index is assigned “not in order” or “not available”, the index is plotted as −1. *P* values are computed using the Wilcoxon signed-rank test (two-sided).

Among the six sets, the overall trends of miRNA targeting bias plots were comparable, except for that of P030 (S2 and S3 Figs). The assessments of (i) avoidance index *A*, (ii) preference index *H*, and (iii) Λ shape demonstrated similar results for both ante-eutherian and eutherian miRNAs among the six sets, except for P030 (Fig 3, S1–S3 Tables); the features (i), (ii), and (iii) also demonstrated that the miRNA targeting specificity is consistently more distinctive for ante-eutherian than for eutherian miRNAs, except for P030.

Apart from the PITA prediction with the highest stringency (P030), the series of assessments showed that the observed miRNA targeting specificity was reproducible across different target prediction methods and various levels of stringency.

### The effect of miRNA repression is modest with paradoxical outcomes

The observed avoidance of miRNA targeting of highly expressed genes may be alternatively interpreted as a consequence of the most highly expressed genes being repressed by the targeting of the most highly expressed miRNAs. To examine this in more detail, we estimated the extent of repression induced by the most highly expressed miRNAs. We traced each of the targeted genes to assess how much its expression intensity is reduced under the condition (organ) in which the targeting miRNA has the highest expression (β condition), compared with that under the condition in which the targeting miRNA is mute (α condition). Of the 1426 miRNAs, we identified 58 that exhibited the β condition in at least one organ and also exhibited the α condition in at least one other organ. We then examined the miRNA–mRNA target pairs, each of which consisted of one of the 58 miRNAs and one of the target genes of the miRNA. For each pair, we compared expression levels of the targeted gene between α and β conditions (see Materials and Methods). Note that, for this examination, we adopted the 11-level grading of gene expression intensity across the 10 organs.

**Fig 4:**
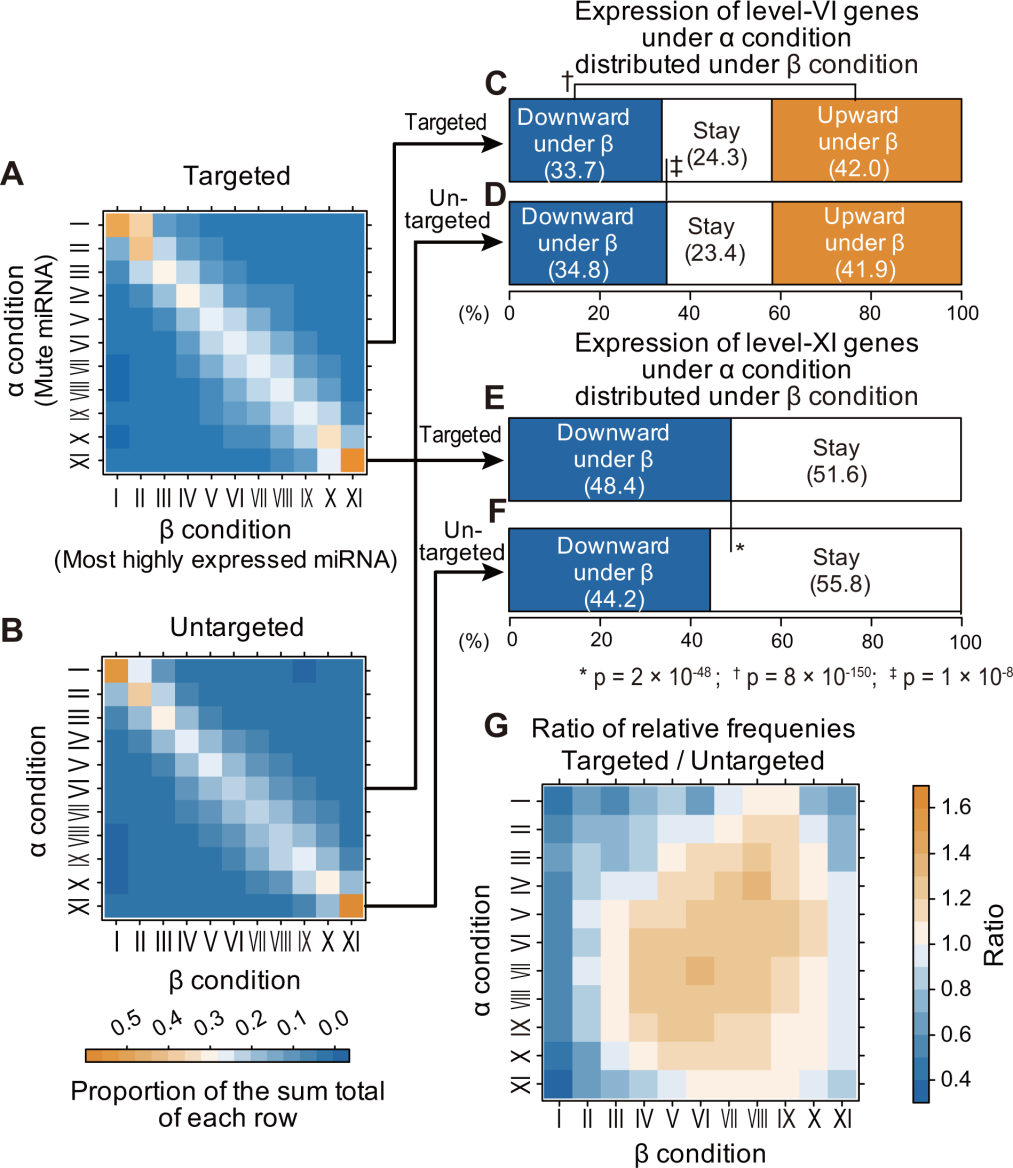
Comparison of target gene expression intensity between conditions of mute miRNA and most highly expressed miRNA. (A, B) Each color-coded matrix is constituted so that each row corresponds to the target gene expression level under the α condition, and each column corresponds to that under the β condition; under the α condition, the expression level of the miRNA that targets the gene is mute, while under the β condition, it is at the highest rank. The elements of each row of the matrix (A) indicate how the expression level of the targeted genes is distributed over the expression level under the β condition using its value as a proportion of the sum total of the row. Thus, the diagonal element indicates the fraction of the targeted genes whose expression level is the same between the β condition and the α condition for each row. Matrix (B) shows the relationship of the expression level of the untargeted genes between the α and β conditions in the same way as in (A). (C) Bar chart showing how the targeted genes of level VI under the α condition are distributed over the levels under the β condition with the fractions of three categories: downward shift in expression level under β, here denoted as DSuβ (downward shift under β), staying the same, and upward shift in expression level under β. (D) For the untargeted genes, the bar chart is shown in the same way as in (C). (E) Bar chart indicating how the targeted genes of level XI under the α condition are distributed over the levels under the β condition with the fractions of two categories, namely, DSuβ and staying the same. (F) For the untargeted genes, the bar chart is shown in the same way as in (E). (G) The matrix indicates the ratio of the relative frequency of targeting occurrences for (true) targeted genes to that for (background) untargeted genes. This was computed for each combination of α and β (see also Materials and Methods).

The largest fraction of targeted genes under the α condition (mute miRNA) was shown to maintain the same level of expression even under the β condition (highest miRNA) across all levels of α (Fig 4A, diagonal elements). If the miRNA substantially represses the expression of its target gene, the fraction of targeted genes with downward shifts in expression level under the β condition, here denoted as DSuβ (downward shift under β), would be expected to increase; however, the fraction of targeted genes with DSuβ was instead reduced, as illustrated in the elements on the left side of the diagonal in each row of the plot in Fig 4A. To obtain background distribution levels, we conducted the same analysis on *untargeted* genes (Fig 4B), which showed a distribution similar to that of targeted genes.

We next examined level VI (the intermediate expression level) under the α condition in detail because it has an equal number of degrees of level-shifting either upward (VII–XI, rightward of the row) or downward (I–V, leftward) under β. Despite the repression effect, the fraction of targeted genes with DSuβ (33.7%) was significantly smaller than that of targeted genes with an upward shift in expression level under β, denoted as USuβ (upward shift under β) (42.0%) (*P* = 8 × 10^−150^) (Fig 4C), and the fraction of targeted genes with DSuβ was significantly smaller than that of untargeted genes with Duβ (34.8%) (*P* = 2 × 10^−8^) (Fig 4D). These results were paradoxical in two ways; i) a larger number of targeted genes were up-regulated rather than repressed, and ii) untargeted genes were down-regluated more often rather than targeted, which suggested that the effect of miRNA repression is modest.

### miRNA repression does not play major roles in forming the intermediate level of gene expression

The most highly expressed level XI under α can be either maintained or shifted downward under β; of the two alternatives, the fraction of targeted genes with DSuβ (48,4%) (Fig 4E) was larger than that of untargeted genes with DSuβ (44.2%) (Fig 4F). Therefore, the four-percentage-point difference (*P* = 4 × 10^−48^) can be attributable to the repression effect. This indicated that although the fraction of target genes on which substantial repression is exerted is small, the repression effect on the most highly expressed target genes is consistently significant.

To determine whether the observed repression effect contributes to forming the intermediate level of target gene expression, we explored which α-β level combination fits best with miRNA targeting. To this end, we examined how often *true* miRNA targeting occurs compared with *background* targeting for untargeted genes using the ratio of their relative frequencies. The ratio matrix (Fig 4G) showed the optimal combinations as two peaks at (α = IV and β = VIII) and (α = VII and β = VI). This result indicated that regardless of the presence or absence of actual targeting, target genes maintain intermediate levels of expression; furthermore the former peak illustrates the paradoxical upregulation of the target genes in spite of the targeting miRNAs’ presence. (note that miRNAs under the α condition lack targeting because they are not expressed.) These results suggest that miRNA repression does not play a major role in causing an intermediate level of target gene expression.

### Cross-species and -platform reproducibility

To investigate whether the miRNA targeting specificities can be observed other species than human, we examined mouse by-organ expression profiles of miRNA precursors [15] and protein-coding genes [20]. We identified 149 mouse miRNA orthologs to human and examined them by applying our methods (see details in S1 Text). As a result, we found that the patterns of mouse miRNA targeting were consistent to those of human such that the Λ shape was observed (S6 Fig and S4 Table) and targeting preference (*H*) (S5 Table) and avoidance (*A*) (S6 Table) indices were higher for old Ante-eutherian than Eutherian miRNAs; although the degree of distinctiveness of the pattern was reduced probably due to a smaller number of mouse miRNA orthologs available (149) than that of miRNAs used for human (1426).

To check cross-platform reproducibility, we used a microarray-based miRNA expression data set [21] together with the high-throughput-sequencing (HTS)-based profile of protein-coding genes [20] (see details in S1 Text). Applying our methods to the pair of profiles, we confirmed that the patters of miRNA targeting specificities were robustly reproduced (S8 Fig); such that the number of Λ-shapes (S7 Table), the preference (*H*) (S8 Table) and avoidance (*A*) (S9 Table) indices (S9 Fig) were significantly greater for old Ante-eutherian than for Eutherian miRNAs.

## Discussion

We herein demonstrated that miRNAs preferentially target genes of intermediate expression levels while avoiding highly expressed ones, and that this specificity is more pronounced for evolutionarily older miRNAs, suggesting that it has been shaped by evolution. Consistent with the finding that animal miRNAs do not cleave many mRNAs under physiological conditions [22,23], our results showed that the effect of repression by miRNAs is modest or rather often masked by regulatory effects occurring in the opposite direction. Therefore, the observed miRNA targeting specificity is not a direct consequence of the repression.

We also found that the relationship of miRNA–mRNA expression intensities largely determines the miRNA targeting preference, without regard for the target function; this may explain why animal miRNAs have targets with such a diverse range of functions [12]. It is important that the miRNA targeting specificity and its more distinctive delineation for the evolutionarily older miRNAs were consistently shown for human and also mouse; because these results raise the possibility that the targeting specificity we found is shared in mammals. We also confirmed the cross-platform reproducibility by using a microarray-based data set in addition to the series of RNA-seq ones, which supports the robustness of the analytical methods we used.

Our results captured that highly expressed miRNAs indeed repress the highly expressed target genes; but when seeing the intermediately expressed genes, a larger number of such target genes were paradoxically upregulated rather than repressed even in the high-level presence of miRNAs. Those miRNAs that target the intermediate-level genes may superficially be interpreted as exerting ‘tuning interactions’ because they seem to interact with many genes modulated in the probably optimal intermediate range; however, actually, our results implicated that the interactions include a considerable number of dispensable ones.

This may be consistent with the possibility that many miRNAs’ targeting are neutral (tolerated/buffered) rather than tuning interactions [2,3]. The dispensable facet of miRNA interactions coincides with the finding that an in-vivo study of miRNA inhibition by ‘antagomirs’ showed a substantial number of the targets not being derepressed (upregulated) but paradoxically downregulated [24]. On the other hand, recently, many abnormal knockout phenotypes were reported for the mouse broadly conserved miRNA families (reviewed in [25]), the member miRNAs of which correspond basically to Ante-Eutherian miRNAs of our study. Each of such miRNA genes is, indeed, indispensable. However, knocking out one miRNA gene disrupts all of its targeting interactions, even if the specific abnormality is caused by the disruption of one targeting interaction [26], therefore there remains the possibility that the miRNA targeting interactions include a considerable number of dispensable ones. Our study, in particular, delineated that the dispensable or neutral miRNA interactions are enriched in targeting the intermediately expressed genes.

## Materials and methods

### Human 3′ UTR sequences

Sequences and annotations of the human protein-coding transcripts registered in Ensembl 89 were downloaded through BioMart (http://www.ensembl.org/). The transcripts and genes assigned to chromosomes 1–22, X and Y were retained. When multiple transcripts were annotated for a gene, a representative transcript was chosen for each gene using the following criteria in decreasing order of priority: i) longer CDS (coding sequence), ii) a coding end located further downstream, iii) longer 3′ UTR, and iv) longer transcript. A total of 18,951 loci were assigned to single transcripts.

### Acquisition of human miRNA information

Information about human miRNAs was obtained from miRBase release 21 by downloading file ‘miRNA. dat’ (ftp://mirbase.org/pub/mirbase/21/). Annotations and sequences corresponding to 1426 precursor miRNAs, for which we previously identified their time of origin [6], were extracted. Note that our previous study reported 1433 precursor miRNAs based on release 18; after this, two precursor miRNA entries were removed in release 19: hsa-mir-720 and hsa-mir-4482-1; then five precursor entries hsa-mir-3118-6, -3669, -3673, -511-2, -1686 were removed in release 21 compared to release 19. The annotations and sequences of 1936 mature miRNAs linked to the 1426 precursor miRNAs by database record descriptions were also extracted.

### miRNA target prediction by TargetScan

Of the 19,021 representative transcripts, 3′ UTR sequences of 8 bp or longer were retrieved (n = 18,951), and the corresponding 3′ UTR sequences were excised from the human genome sequence of Ensembl GRCh37.89, which are named here as the 3′ UTR set. TargetScan 7.01 was used to search the sequences of the 3′ UTR set for the target sites of 1936 mature miRNAs, which corresponded to 1426 precursor miRNAs. For the hits, TargetScan CSs were computed by targetscan_70_context_score.pl (version 7.01) using default settings. Every hit was retained that had an exact match with a 7-mer seed sequence or longer (i.e., 7mer-m8 and 8mer-1a in the program’s terminology) and CS < 0. Overlapping hit sites for a mature miRNA within a gene were made into a single nonredundant site as described below.

Hit sites with overlaps removed were sorted by the CSs into three sets with the following CS thresholds: (i) < −0.4, (ii) < −0.5, and (iii) < −0.6. Each of the three sets was converted into a matrix of 18,951 rows (genes) × 1936 columns (mature miRNAs), named (i) C010, (ii) C020, and (iii) C030, respectively; the matrices were generically named ***g2m***_[*g*, *μ*]_, where each element represents the number of predicted target sites of a mature miRNA *μ* in a gene *g*.

### miRNA target prediction by PITA

To accurately calculate the thermodynamic accessibility of miRNAs to the target site at the 5′ or 3′ end of a 3′ UTR, the coding or poly-A sequence flanking the 3′ UTR was required, respectively. Thus, the 25-nt coding sequences immediately upstream of the 3′ UTRs and 15-nt consecutive adenines were concatenated to the 5′ and 3′ ends of each 3′ UTR sequence, respectively, which is named the flank-added 3′ UTR in this study. A PITA search was conducted on the flank-added 3′ UTRs with the following parameters: flank_up 3, flank_down 15, and remaining default settings. Every hit was retained for which the whole seed-matched sequence was located within the 3′ UTR and was 7-mer or longer, excluding wobbled pairing (i.e. 7:0:0 and 8:0:0 in the program’s terminology).

Overlapping hit sites were made into a single nonredundant site in the same way as the TargetScan prediction. Then, the resultant hits were sorted by the free energy of the accessibility *ΔΔG* in decreasing order and grouped into three sets, such that each set had the closest number of hits to that of the TargetScan hits of (i) C010, (ii) C020, and (iii) C030, which corresponded to the ranges (i) *ΔΔG* < -0.96, (ii) < −3.69, and (iii) < −9.49, respectively. Each of the three sets was converted to a 18,951 × 1936 matrix and named (i) P010, (ii) P020, and (iii) P030, respectively, generically termed ***g2m*** as in the C0X0 series. *ΔΔG* was computed by PITA by subtracting the free energy lost by unpairing the target site (*Δ*_*Gopen*_) from the free energy gained by binding of the miRNA to its target (*ΔG*_*duplex*_) [19].

### Relationship of mature, precursor miRNAs and target genes

A precursor (or hairpin) miRNA yields a maximum of two types of mature miRNA (3p and 5p arms of the precursor), although in some cases multiple distinct precursors yield one identical mature miRNA as annotated by miRBase database records. For simplicity, the relationship of the precursor with the mature miRNA was represented by a 1936 × 1426 matrix, ***m2h***, which associates each mature miRNA with its precursor(s): ***m2h***_[*μ*, *h*]_ = 1 when the *μ*th mature miRNA is yielded from the *h*th precursor; otherwise ***m2h***_[*μ*, *h*]_ = 0. The relationship of precursors to target genes was then given by ***1***_*A*_(***g2m***·***m2h***), which yields a 1/0 matrix of 18,951 × 1426, named ***G2H***, where ***1***_*A*_ is an indicator function: ***1***_*A*_(*x*) = 1 if *x* > 0, ***1***_*A*_(*x*) = 0 if *x* = 0, because both ***g2m*** and ***m2h*** consist of non-negative integers.

Therefore, the matrix ***G2H***_[*g*,*h*]_ connects a precursor to its target genes bridging the intermediate mature miRNAs. ***G2H***_[*g*,*h*]_ = 1 if the *h*th precursor yields mature miRNA(s) and any of the mature miRNA(s) target the *g*th gene; otherwise, ***G2H***_[*g*,*h*]_ = 0, namely, the cases where the *h*th precursor yields mature miRNA(s) and none of the mature miRNA(s) targets the *g*th gene. ***G2H*** means that even if a gene has multiple target sites from a mature miRNA, it is regarded as 1, and if a mature miRNA that is derived from multiple precursors has a target gene, all of the precursors from which it is derived are assigned 1.

### Expression intensity of protein-coding genes

To obtain the expression intensity of human protein-coding genes by organ, “S1 Table” in the paper by Uhlén et al. [16] was downloaded. The normalized read counts, and FPKM (fragments per kilobase of exon per million mapped fragments) of every gene whose ENSG number was included in the 18,951 genes of interest were selected. The read counts for the brain, heart, kidney, liver, ovary, pancreas, prostate, thyroid, placenta, and testis were retrieved.

### Expression intensity of miRNAs

The RNA-seq profiles of human miRNA precursors (“S9 Table” in mmc10.xls of the paper by Landgraf et al. 2007 [15]) were downloaded, in which the counts of cloned sequences were mapped to the human precursor miRNA. Note that the precursor corresponds uniquely to the miRNA locus. Those cloned sequences that exactly matched human genome sequences of the miRNA loci were retrieved. The precursors whose names matched the 1426 precursors of interest were retained. For a miRNA locus that had both the 3p and the 5p clones sequenced, the larger clone count was used.

Clone counts were available for the samples of 36 normal human organs/tissues. From these 36, the following 10 organs that were included in the dataset of protein-coding genes reported by Uhlén *et al*. [16] were selected: brain, heart, kidney, liver, ovary, pancreas, prostate, thyroid, placenta, and testis. Clone counts of the frontal cortex, midbrain, and hippocampus were averaged to obtain a clone count for the brain compatible with that of the expression of protein-coding genes.

### miRNA targeting biases *B* and *B′*

According to the expression intensity in each organ, the 1426 miRNAs were binned into five ranks such that rank 1 included every mute miRNA (i.e., with no expression in that organ but with detectable expression in at least one other organ), and ranks 2–5 were arranged as equally populated bins in increasing order of expression intensity. Likewise, the 18,951 protein-coding genes were binned into 11 levels (I–XI); level I was assigned for all mute genes, and levels II–XI were arranged as equally populated in increasing order of expression intensity.

In organ *z* | *z* ∈ {1, 2,…, 10} for the miRNA age or time of origin *t* | *t* ∈ {1, 2, 3, 4}, we enumerated every occurrence of a miRNA of rank *j* | *j* ∈ {1, 2, 3, 4, 5} targeting a gene of level *l* | *l* ∈ {1, 2,…, 11}, and denoted the number of occurrences as *o*_*l*,*j*,*z*,*t*_. We also made five-rank grading bins *i* | *i* ∈ {1, 2, 3, 4, 5}) of the 18,951 genes, where the original 11 levels of grading were converted into the five ranks so that the number of occurrences of level 1 (mute) genes was reassigned to that of rank 1. The numbers of occurrences of levels II–IV, V–VII, and VIII–X genes were each averaged and reassigned to those of ranks 2, 3, and 4, respectively, and the number of occurrences of level X1 (highest) genes was reassigned to that of rank 5. The number of occurrences converted to those of the five-rank grading was denoted as capital *O*_*i*,*j*,*z*,*t*_ (S5 Fig). The 11-level to five-rank conversion was performed to smooth the plot presentations.

A contingency table was then made in which each element indicates the number of targeting occurrences *O*_*i*,*j*,*z*,*t*_, with the rank of gene expression intensity in the row and the rank of miRNA expression intensity in the column. Assuming independence between the expression intensity of miRNAs and that of their coexpressed targeted genes, the expected probability *P*_*i*,*j*,*z*,*t*_ of a rank-*j* miRNA targeting a rank-*i* gene is given as the product of the marginal probability of rank *j*’s miRNAs targeting genes and the marginal probability of rank *i*’s genes being targeted, as *P*_*i*,*j*,*z*,*t*_ = (Σ^5^_*j* = 1_ *O*_*i*,*j*,*z*,*t*_/W_*z*,*t*_) × (Σ^5^_*i* = 1_ *O*_*i*,*j*,*z*,*t*_/W_*z*,*t*_), where W_*z*,*t*_ = Σ^5^_*i* = 1_Σ^5^_*j* = 1_ *O*_*i*,*j*,*z*,*t*_. Then the expected occurrence *E*_*i*,*j*,*z*,*t*_ is computed as *E*_*i*,*j*,*r*,*t*_ = *P*_*i*,*j*,*z*,*t*_ ×W_*z*,*t*_.

We evaluated how much the observed *O*_*i*,*j*,*z*,*t*_ deviated from the expected *E*_*i*,*j*,*z*,*t*_ with the bias *B*_*i*,*j*,*z*,*t*_ using Pearson’s chi-squared test statistic (*C*_*i*,*j*,*z*,*t*_) with its log_10_ p value being signed plus when larger than expected and minus when less, as follows:

*B*_*i*,*j*,*z*,*t*_ = −log_10_ {*Χ*^2^_*ν* = 1_ (*X* > *C*_*i*,*j*,*z*,*t*_)} if *O*_*i*,*j*,*z*,*t*_ ≥ *E*_*i*,*j*,*z*,*t*_, otherwise *B*_*i*,*j*,*z*,*t*_ = log_10_ {*Χ*^2^_*ν* = 1_ (*X* > *C*_*i*,*j*,*z*,*t*_)}, where *C*_*i*,*j*,*z*,*t*_ = (*O*_*i*,*j*,*z*,*t*_ − *E*_*i*,*j*,*z*,*t*_)^2^/ *E*_*i*,*j*,*z*,*t*_. In the same way, we computed the bias *B′*_*l*,*j*,*z*,*t*_ based on the 11-level grading *l* ∈ {1, 2,…, 11} of the gene expression intensity.

### Avoidance index *A*

The avoidance index, *A*_*z*,*t*_, is computed as the sum of the extent of avoidance of the most highly expressed miRNAs against the most highly expressed target genes *C*_5,5,*z*,*t*_ and that of preference of mute miRNAs *C*_5,1,*z*,*t*_. We regarded the avoidance index *A*_*z*,*t*_ as “not in order” unless *B*_5,1,*z*,*t*_ (the blue line at the right-hand side of the plot) was > 0 and *B*_5,5,*z*,*t*_ (the red line at the right-hand side of the plot) was < 0 (Fig 2E). It is formally written as *A*_*r*,*t*_ = *C*_5,5,*z*,*t*_ + *C*_5,1,*z*,*t*_ if (*B*_5,1,*z*,*t*_ > 0) ∩ (*B*_5,5,*z*,*t*_ < 0), but “not in order” otherwise.

### Criterion of Λ shape of targeting preference

We defined the upward convexity, Λ, in the plot of the bias using the criterion that the minimum value of the bias of the most highly expressed miRNAs to the target genes of ranks 2–4 (intermediate expression) should be larger than the biases to both ranks 1 and 5 of the target genes (Fig 2E), as Λ if min_*i* = 2,3,4_ (*B*_*i*,5,*z*,*t*_) > max_*i* = 1,5_ (*B*_*i*,5,*z*,*t*_). Similarly, the downward convexity, V, is determined if max_*i* = 2,3,4_ (*B*_*i*,5,*z*,*t*_) < min_*i* = 1,5_ (*B*_*i*,5,*z*,*t*_).

### Preference index *H*

The height of the upward convexity was quantified as the preference index (*H*) (Fig 2E) with the sum of (i) the chi-square statistic value for the maximum bias of the intermediate-expression ranks of the target genes *C*_i*,5,*z*,*t*_ | i^*^ = argmax_*i* = 2,3,4_ (*B*_*i*,5,*z*,*t*_), and (ii) that for the larger bias of either the mute or the most highly expressed ranks of the target genes *C*_i*,5,*z*,*t*_ | i^*^ = argmax_*i* = 1,5_ (*B*_*i*,5,*z*,*t*_). The maximum bias to genes of intermediate expression is required to be positive and the maximum bias to the mute and most highly expressed ranks is required to be negative. Thus, *H*_*z*,*t*_ = *H*_rise_ + *H*_dive_ if (max_*i* = 2,3,4_ (*B*_*i*,5,*z*,*t*_) > 0) ∩ (max_*i* = 1,5_ (*B*_*i*,5,*z*,*t*_) < 0), but “not in order” otherwise, where *H*_rise_ = *C*_i*,5,*z*,*t*_ for i^*^ = argmax_*i* = 2,3,4_ (*B*_*i*,5,*z*,*t*_), and *H*_dive_ = *C*_i*,5,*z*,*t*_ for i^*^ = argmax_*i* = 1,5_ (*B*_*i*,5,*z*,*t*_).

### Test for the avoidance and preference indices between ante-eutherian and eutherian miRNAs

The indices of avoidance *A*_*z*,*t*_ and preference *H*_*z*,*t*_ were compared between miRNAs with ante-eutherian (*t* = 1) and eutherian origins (*t* = 2) for the 10 organs *z* ∈ {1, 2, …, 10}. *A*_*r*,*t*_ is “not in order” unless (*B*_5,1,*z*,*t*_ > 0) ∩ (*B*_5,5,*z*,*t*_ < 0). *H*_*r*,*t*_ is “not in order” unless (max_*i* = 2,3,4_ (*B*_*i*,5,*z*,*t*_) > 0) ∩ (max_*i* = 1,5_ (*B*_*i*,5,*z*,*t*_) < 0). We regarded “not in order” as comparable to the lowest value of the indices, which was thus assigned pseudo-value 0. The indices *A*_*z*,1_ and *A*_*z*,2_ were tested by the Wilcoxon signed-rank test (two-sided), where if either *A*_*z*,1_ or *A*_*z*,2_ but not both is “not in order” then *A*_*r*,*t*_ = 0; if both *A*_*z*,1_ and *A*_*z*,2_ are “not in order”, then both *A*_*z*,*t*_s were excluded from the test. *H*_*z*,1_ and *H*_*z*,2_ were tested in the same way.

### Estimation of the effect of repression

The multiset *D* consists of the ordered tuples *d*s, of which each tuple *d* is a pair of levels *α* ∈ {1,2,…, 11} and *β* ∈ {1,2,…, 11}, such that:

$$D=\left\{d=\left(\alpha ,\beta \right)=\left(R_{\left(g,z^{\prime }\right)},R_{\left(g,z^{\prime \prime }\right)}\right)|T_{\left(\mu ,g\right)}=1\cap \left[\exists z^{\prime }\in z\;\exists z^{\prime \prime }\in z\;\left(\rho_{\left(\mu ,z^{\prime }\right)}=1\right)\cap \left(\rho_{\left(\mu ,z^{\prime \prime }\right)}=5\right)\right]\right\}$$ 1

where *g*, *μ*, and *z* stand for a gene, a miRNA, and an organ of interest *g* ∈ {1, 2,…, 18,951}, *μ* ∈ {1, 2,…, 1426}, and *z* ∈ {1, 2, …, 10}, respectively. Function *R* gives the level *R*_(*g*, *z*)_ → {1, 2, …, 11} of the expression of gene *g* in an organ *z*, and function *ρ* gives the rank *ρ*_(*μ*, *z*)_ → {1, 2, 3, 4, 5} of a miRNA *μ* in an organ *z*. The indicator function *T*_(*μ*, *g*)_ = 1 if a miRNA *μ* targets a gene *g*, or 0 otherwise. In this analysis, the expression levels were assigned across all organs but not in each organ because different organs were compared for expression levels.

The multiplicity of an ordered tuple *d =* (*α*, *β*) of the multiset *D* is denoted as *m*_*D*_(*d*). The value of the element of row *α* and column *β* of the matrix ***Δ***_[α,β]_ is given as

*δ*_[*α*,*β*]_ = *m*_*D*_(*d*). The matrix ***Δ*** was then transformed so that the sum of each row is 1, which was named ***Δ*′** as *δ*′_[*α*, *β*]_ = *δ*_[*α*, *β*]_/Σ_*β*_ (*δ*_[*α*, *β*]_). As the background, the multiset *D* and the ordered tuple *d* are given by simply making *T*_(*μ*, *g*)_ = 0, which is changed from *T*_(*μ*, *g*)_ = 1 as follows:

$$D=\left\{d=\left(\alpha ,\beta \right)=\left(R_{\left(g,z^{\prime }\right)},R_{\left(g,z^{\prime \prime }\right)}\right)|T_{\left(\mu ,g\right)}=0\cap \left[\exists z^{\prime }\in z\;\exists z^{\prime \prime }\in z\;\left(\rho_{\left(\mu ,z^{\prime }\right)}=1\right)\cap \left(\rho_{\left(\mu ,z^{\prime \prime }\right)}=5\right)\right]\right\}$$ 2

***Δ*** and ***Δ*****′** are given from *d* and *D*, in the same way as ***Δ*** and ***Δ*′**; additionally, the ratio *k*_[*α*, *β*]_ of relative frequency of targeting occurrences for *true* targeted genes *δ*_[*α*, *β*]_/Σ_*α*_Σ_*β*_ (*δ*_[*α*, *β*]_) to that for *background* untargeted genes *δ*′_[*α*, *β*]_/Σ_*α*_Σ_*β*_ (*δ*′_[*α*, *β*]_) was computed for each combination of α and β. (Fig 4A, 4B, and 4G depict color-coded presentations of matrices ***Δ*′**, ***Δ*****′**, and ***K***, respectively).

### Removal of overlapping hit sites

When two target sites overlap, the target site that starts from a more 5′ position is retained and the other is removed. This rule of removal proceeds in a stepwise manner from the 5′ end of the 3′ UTR sequence to the 3′ end. As a consequence, in the case of multiple overlapping target sites arranged in a chain, the bridging target site is removed and the others are retained; in the case of multiple stacked overlaps, only the most 5′ site is retained (see S5 Fig).

## Supporting information

S1 Fig miRNA targeting bias plotted by applying the 11-level grading contingency table. All plots are drawn based on 11-level grading contingency tables; hence, the x-axis has 11 ticks according to the expression intensity of the genes. In each panel, the bias of how the observed miRNA targeting occurrences deviates from expected occurrences is plotted against the expression levels of target genes for the most highly expressed (red) and mute (blue) miRNAs of (A) ante-eutherian origin and (B) eutherian origin.
(EPS)

S2 Fig Comparison of miRNA targeting bias plots using different prediction methods and stringencies based on the five-rank grading contingency table. All plots are drawn based on the five-rank grading contingency tables; hence, the x-axis has five ticks according to the expression intensity of the genes. In each panel, the bias of how the observed number of miRNA targeting occurrences deviates from expected is plotted against the expression levels of the target genes for the most highly expressed (red) and mute (blue) miRNAs. Ante and Euth stand for ante-eutherian and eutherian origins of miRNAs, respectively. (A–C) Based on TargetScan-predicted sets of miRNA target sites with an increasing order of stringency, each of which is named C010, C020, and C030, a comparison of miRNA targeting bias is shown between old Ante (upper row) and younger Euth (lower row) miRNAs. (D–F) Based on PITA-predicted sets of miRNA target sites with an increasing order of stringency, each of which is named P010, P020, and P030, a comparison of miRNA targeting bias is shown between old Ante (upper row) and younger Euth (lower row) miRNAs. The PITA stringency is configured so that each P0X0 has the nearest number of predicted target sites to that of the corresponding C0X0.
(EPS)

S3 Fig Comparison of miRNA targeting bias using different prediction methods and stringencies based on the 11-level grading contingency table. By applying the 11-level contingency table, all plots are drawn the same way as in S2 Fig. In each panel, the bias of how the observed number of miRNA targeting occurrences deviates from expected is plotted against the expression levels of target genes for the most highly expressed (red) and mute (blue) miRNAs. Ante and Euth stand for ante-eutherian and eutherian origins of miRNAs, respectively. (A–C) Based on TargetScan-predicted sets of miRNA target sites with increasing levels of stringency, each of which is named C010, C020, and C030, the miRNA targeting bias is shown in comparison between old Ante (upper row) and younger Euth (lower row) miRNAs. (D–F) Based on PITA-predicted sets of miRNA target sites with increasing level of stringency, each of which is named P010, P020, and P030, the miRNA targeting bias is shown in comparison between old Ante (upper row) and younger Euth (lower row) miRNAs. The PITA stringency is configured so that each P0X0 has the nearest number of predicted target sites to that of the corresponding C0X0.
(EPS)

S4 Fig Conversion of contingency table from 11-level to five-rank. Contingency table (left) of the observed numbers of miRNA targeting occurrences in which the 11 levels (I–XI) of expression intensity of the genes are arranged in rows and the five ranks (1–5) of the miRNA expression intensity in columns. Level I includes every mute gene, and levels II–XI are arranged as equally populated in increasing order of the expression intensity of the genes. Likewise, for miRNAs, rank 1 includes every mute miRNA, and ranks 2–5 are equally populated in increasing order. The 11-level grading contingency table is converted into the five-rank grading contingency table (right) so that the observed numbers of occurrences of levels II–IV, V–VII, and VIII–X are each averaged to those of ranks 2, 3, and 4, respectively, while those of levels I and XI become ranks 1 and 5, respectively. The numbers of the elements of the two contingency tables represent the case where the brain is the organ and the miRNA time of origin is ante-eutherian.
(EPS)

S5 Fig Removal of overlapping target sites. (A) When two target sites (rectangles) overlap within the 3′ UTR sequence (horizontal bar), we retained the target site at the most 5′ position (solid-line rectangle) and removed the other (broken-line rectangle). This proceeded in a stepwise fashion from the 5′ end to the 3′ end of the 3′ UTR sequence. As a consequence, in the case of multiple overlapping target sites arranged in a chain (B), the bridging target site was removed, while the others were retained. (C) In the case of multiple stacked overlaps, only the most 5′ site was retained.
(EPS)

S6 Fig Mouse miRNA targeting specificity. In the same way as Fig 2C and 2D, the bias reflecting how the observed number of miRNA targeting occurrences deviates from the expected number is plotted against the five expression ranks of target genes for the most highly expressed (red) and mute (blue) miRNAs in each organ by using a mouse data set. The bias is indicated using Pearson’s chi-squared test statistic with its log_10_ p value being signed plus when larger than expected and minus when less. Plots of the targeting bias are drawn for the miRNAs of ante-eutherian origin (upper row) and eutherian origin (lower) for the 7 organs.
(EPS)

S7 Fig Targeting specificities are more evident for older miRNAs by a mouse data set. Ante and Euth stand for ante-eutherian and eutherian origins of miRNAs, respectively. In the upper row, based on TargetScan-predicted sets of miRNA target sites of mouse with an increasing level of stringency, named C010, C020, and C030, the extent of miRNA targeting avoidance (avoidance index *A*) is compared between Ante (old) and Euth (younger) miRNAs. In the lower row, targetScan-predicted mouse sets of C010, C020, and C030 are applied to compute preference index *H* and compare *H* between Ante and Euth miRNAs. For every plot, when the index is assigned “not in order” or “not available”, the index is plotted as −1.
(EPS)

S8 Fig miRNA targeting specificity by a microarray-based miRNA expression data set. In the same way as Fig 2C and 2D, the bias reflecting how the observed number of miRNA targeting occurrences deviates from the expected number is plotted against the five expression ranks of target genes for the most highly expressed (red) and mute (blue) miRNAs in each organ by using a microarray-based data set. The bias is indicated using Pearson’s chi-squared test statistic with its log_10_ p value being signed plus when larger than expected and minus when less. Plots of the targeting bias are drawn for the miRNAs of ante-eutherian origin (upper row) and eutherian origin (lower row) for the 6 organs.
(EPS)

S9 Fig Targeting specificities are more evident for older miRNAs by a microarray-based miRNA expression data set. In the same way as Fig 3, the plots are made based on a microarray-based data set of by-organ human miRNA expressions. Ante and Euth stand for ante-eutherian and eutherian origins of miRNAs, respectively. (A–C) Based on TargetScan-predicted sets of miRNA target sites with an increasing level of stringency, named C010, C020, and C030, the extent of miRNA targeting avoidance (avoidance index *A*) is compared between Ante (old) and Euth (younger) miRNAs. (D–F) Based on PITA-predicted sets of target sites with an increasing level of stringency, named P010, P020, and P030, the avoidance index *A* is compared between Ante and Euth miRNAs. (G–I) TargetScan-predicted mouse sets of C010, C020, and C030 are applied to compute preference index *H* and compare *H* between Ante and Euth miRNAs. (J–L) PITA-predicted sets of P010, P020, and P030 are applied to compare the preference index *H* between Ante and Euth miRNAs. For every plot, when the index is assigned “not in order” or “not available”, the index is plotted as −1.
(EPS)

S1 Table Λ shape in the plots under Various Conditions of miRNA target prediction. Λ represents upward convexity in the plot of the bias of the most highly expressed miRNA that meets the criterion of the Λ shape. The upward convexity indicates that the miRNAs preferentially target genes with intermediate expression. Hyphens indicate a non-Λ-shaped plot or not available. Ante and Euth represent ante-eutherian and eutherian origins of miRNAs, respectively. C010, C020, and C030 are the sets of predicted target sites by TargetScan Context++ Score in increasing order of stringency; P010, P020, and P030 are those predicted by PITA, so that each of them has the nearest number of target sites to that of C0X0 sets. Br, Brain; He, Heart; Ki, Kidney; Li, Liver; Ov, Ovary; Pa, Pancreas; Pr, Prostate; Th, Thyroid; Pl, Placenta; Te, Testis.
(DOCX)

S2 Table Preference index *A* under Various Conditions of miRNA target prediction. Ante and Euth represent ante-eutherian and eutherian origins of miRNAs, respectively. Hyphens indicate not available or “not in order”. C010, C020, and C030 are the sets of predicted target sites by TargetScan Context++ Score in increasing order of stringency; P010, P020, and P030 are those predicted by PITA, so that each of them has the nearest number of target sites to that of C0X0 sets. Br, Brain; He, Heart; Ki, Kidney; Li, Liver; Ov, Ovary; Pa, Pancreas; Pr, Prostate; Th, Thyroid; Pl, Placenta; Te, Testis. *Derived using Wilcoxon signed-rank test (two-sided; see Materials and Methods).
(DOCX)

S3 Table Preference index *H* under Various Conditions of miRNA target prediction. Ante and Euth represent ante-eutherian and eutherian origins of miRNAs, respectively. Hyphens indicate not available or “not in order”. C010, C020, and C030 are the sets of predicted target sites by TargetScan Context++ Score in increasing order of stringency; P010, P020, and P030 are those predicted by PITA, so that each of them has the nearest number of target sites to that of C0X0 sets. Br, Brain; He, Heart; Ki, Kidney; Li, Liver; Ov, Ovary; Pa, Pancreas; Pr, Prostate; Th, Thyroid; Pl, Placenta; Te, Testis. *Derived from Wilcoxon signed-rank test (two-sided; see Materials and Methods).
(DOCX)

S4 Table Λ shape in the plots of mouse miRNAs. Λ and V represent upward and downward convexities in the plot of the bias of the most highly expressed miRNAs that meet the criterion of the Λ and V shapes, respectively. The upward convexity indicates that the miRNAs preferentially target genes with intermediate expression. Hyphens indicate a non-Λ-non-V-shaped plot or not available. Ante and Euth represent ante-eutherian and eutherian origins of miRNAs, respectively. C010, C020, and C030 are the mouse sets of predicted target sites by TargetScan Context++ Score in increasing order of stringency. Br, Brain; He, Heart; Ki, Kidney; Li, Liver; Ov, Ovary; Pl, Placenta; Te, Testis.
(DOCX)

S5 Table Preference index *A* under Various Conditions of mouse miRNA target prediction. Ante and Euth represent ante-eutherian and eutherian origins of miRNAs, respectively. Hyphens indicate not available or “not in order”. C010, C020, and C030 are the mouse sets of predicted target sites by TargetScan Context++ Score in increasing order of stringency. *Derived from Wilcoxon signed-rank test (two-sided; see Materials and Methods) attesting different *A* between Ante and Euth over the mouse C0X0 matrix series as a whole. Br, Brain; He, Heart; Ki, Kidney; Li, Liver; Ov, Ovary; Pl, Placenta; Te, Testis.
(DOCX)

S6 Table Preference index *H* under Various Conditions of mouse miRNA target prediction. Ante and Euth represent ante-eutherian and eutherian origins of miRNAs, respectively. Hyphens indicate not available or “not in order”. C010, C020, and C030 are the mouse sets of predicted target sites by TargetScan Context++ Score in increasing order of stringency; P010, P020, and P030 are those predicted by PITA, so that each of them has the nearest number of target sites to that of mouse C0X0 sets. Br, Brain; He, Heart; Ki, Kidney; Li, Liver; Ov, Ovary; Pl, Placenta; Te, Testis. *Derived from Wilcoxon signed-rank test (two-sided; see Materials and Methods) attesting different *A* between Ante and Euth over the mouse C0X0 matrix series as a whole.
(DOCX)

S7 Table Λ shape in the plots by a microarray-based data set under Various Conditions of miRNA target prediction. Λ and V represent upward and downward convexities in the plot of the bias of the most highly expressed miRNAs that meet the criterion of the Λ and V shapes, respectively. The upward convexity indicates that the miRNAs preferentially target genes with intermediate expression. Hyphens indicate a non-Λ-non-V-shaped plot or not available. Ante and Euth represent ante-eutherian and eutherian origins of miRNAs, respectively. C010, C020, and C030 are the sets of predicted target sites by TargetScan Context++ Score in increasing order of stringency; P010, P020, and P030 are those predicted by PITA, so that each of them has the nearest number of target sites to that of C0X0 sets. Br, Brain; Ki, Kidney; Li, Liver; Pa, Pancreas; Th, Thyroid; Te, Testis.
(DOCX)

S8 Table Preference index *A* by a microarray-based data set under Various Conditions of miRNA target prediction. Ante and Euth represent ante-eutherian and eutherian origins of miRNAs, respectively. Hyphens indicate not available or “not in order”. C010, C020, and C030 are the sets of predicted target sites by TargetScan Context++ Score in increasing order of stringency. *Derived from Wilcoxon signed-rank test (two-sided; see Materials and Methods) attesting different *A* between Ante and Euth over each series of C0X0, P0X0 and both^†^ matrices as a whole. Br, Brain; Ki, Kidney; Li, Liver; Pa, Pancreas; Th, Thyroid; Te, Testis.
(DOCX)

S9 Table Preference index *H* by a microarray-based data set under Various Conditions of miRNA yarget prediction. Ante and Euth represent ante-eutherian and eutherian origins of miRNAs, respectively. Hyphens indicate not available or “not in order”. C010, C020, and C030 are the sets of predicted target sites by TargetScan Context++ Score in increasing order of stringency. *Derived from Wilcoxon signed-rank test (two-sided; see Materials and Methods) attesting different *A* between Ante and Euth over each series of C0X0, P0X0 and both^†^ matrices as a whole. Br, Brain; Ki, Kidney; Li, Liver; Pa, Pancreas; Th, Thyroid; Te, Testis.
(DOCX)

S1 Text Supporting methods. (DOCX)
