# Supplementary material for: Human microRNAs preferentially target genes with intermediate levels of expression and its formation by mammalian evolution
Source: PLoS One. 2018 May 24;13(5):e0198142. doi: 10.1371/journal.pone.0198142 (PMC5967834; doi:10.1371/journal.pone.0198142)
Supplement: S2 Table — Ante and Euth represent ante-eutherian and eutherian origins of miRNAs, respectively. Hyphens indicate not available or “not in order”. C010, C020, and C030 are the sets of predicted target sites by TargetScan Context++ Score in increasing order of stringency; P010, P020, and P030 are those predicted by PITA, so that each of them has the nearest number of target sites to that of C0X0 sets. Br, Brain; He, Heart; Ki, Kidney; Li, Liver; Ov, Ovary; Pa, Pancreas; Pr, Prostate; Th, Thyroid; Pl, Placenta; Te, Testis. *Derived using Wilcoxon signed-rank test (two-sided; see Materials and Methods). (DOCX) [file pone.0198142.s011.docx]

| Set | Origin | Br | He | Ki | Li | Ov | Pa | Pr | Th | Pl | Te | p* |
| --- | --- | --- | --- | --- | --- | --- | --- | --- | --- | --- | --- | --- |
| C010 | Ante | 16.0 | 4.9 | 7.6 | 3.7 | 2.5 | 4.7 | - | 0.1 | 6.2 | - | 0.008 |
|  | Euth | - | 0.8 | - | 1.9 | - | 1.2 | - | - | - | - |  |
| C020 | Ante | 16.8 | 4.8 | 7.0 | 3.4 | 7.0 | 13.0 | - | 3.5 | 8.8 | - | 0.008 |
|  | Euth | - | 1.3 | - | 1.6 | 3.9 | 1.2 | 1.4 | - | - | - |  |
| C030 | Ante | 16.4 | 2.2 | 1.7 | 0.9 | 6.8 | 10.3 | - | 2.3 | 12.7 | 0.0 | 0.2 |
|  | Euth | - | 1.1 | 3.4 | 3.6 | 5.1 | 2.0 | 1.4 | - | 1.1 | - |  |
| P010 | Ante | 11.4 | 3.3 | 4.8 | 3.5 | 8.2 | - | - | - | 9.5 | - | 0.03 |
|  | Euth | - | - | - | - | - | - | - | - | - | - |  |
| P020 | Ante | 4.3 | 3.5 | - | 1.1 | 6.8 | - | - | - | 1.4 | - | 0.09 |
|  | Euth | - | - | - | - | - | 1.4 | - | - | - | - |  |
| P030 | Ante | 5.0 | 13.3 | 4.0 | 3.7 | 10.7 | - | - | 5.7 | 1.1 | 1.8 | 0.04 |
|  | Euth | 2.2 | - | 1.7 | - | 2.7 | 3.4 | - | - | 1.1 | - |  |
