# Supplementary material for: Human microRNAs preferentially target genes with intermediate levels of expression and its formation by mammalian evolution
Source: PLoS One. 2018 May 24;13(5):e0198142. doi: 10.1371/journal.pone.0198142 (PMC5967834; doi:10.1371/journal.pone.0198142)
Supplement: S3 Table — Ante and Euth represent ante-eutherian and eutherian origins of miRNAs, respectively. Hyphens indicate not available or “not in order”. C010, C020, and C030 are the sets of predicted target sites by TargetScan Context++ Score in increasing order of stringency; P010, P020, and P030 are those predicted by PITA, so that each of them has the nearest number of target sites to that of C0X0 sets. Br, Brain; He, Heart; Ki, Kidney; Li, Liver; Ov, Ovary; Pa, Pancreas; Pr, Prostate; Th, Thyroid; Pl, Placenta; Te, Testis. *Derived from Wilcoxon signed-rank test (two-sided; see Materials and Methods). (DOCX) [file pone.0198142.s012.docx]

| Set | Origin | Br | He | Ki | Li | Ov | Pa | Pr | Th | Pl | Te | p* |
| --- | --- | --- | --- | --- | --- | --- | --- | --- | --- | --- | --- | --- |
| C010 | Ante | - | 8.2 | 3.0 | 0.9 | 1.2 | 7.1 | 2.1 | 3.7 | 2.7 | - | 0.03 |
|  | Euth | 2.0 | 3.0 | - | 1.6 | - | 2.2 | - | - | - | - |  |
| C020 | Ante | 6.9 | 16.8 | 5.5 | 2.9 | 6.0 | 14.5 | 8.2 | 11.4 | 3.1 | - | 0.004 |
|  | Euth | 6.7 | 4.6 | 2.1 | - | 4.2 | 1.2 | 2.8 | - | - | - |  |
| C030 | Ante | 3.3 | 7.5 | 1.0 | 1.0 | 2.9 | 8.1 | 3.3 | 2.8 | 6.0 | - | 0.3 |
|  | Euth | 3.6 | 3.9 | 3.0 | 0.8 | 6.5 | 1.1 | 3.3 | - | - | - |  |
| P010 | Ante | 5.3 | 5.0 | 2.4 | 1.0 | 3.7 | - | 2.3 | 5.5 | 0.8 | - | 0.008 |
|  | Euth | 0.3 | 4.1 | - | - | - | - | - | - | - | - |  |
| P020 | Ante | 5.1 | 6.4 | - | 1.0 | 6.0 | - | 1.6 | - | 0.6 | - | 0.1 |
|  | Euth | 0.1 | 2.7 | - | - | - | 1.5 | 0.8 | - | - | - |  |
| P030 | Ante | 5.1 | 12.7 | 3.5 | 4.0 | 7.2 | 0.4 | 1.7 | - | 1.8 | 0.6 | 0.25 |
|  | Euth | 1.3 | 3.9 | 2.6 | - | 3.8 | 2.3 | 2.7 | 0.9 | 2.5 | - |  |
