# Supplementary material for: Human microRNAs preferentially target genes with intermediate levels of expression and its formation by mammalian evolution
Source: PLoS One. 2018 May 24;13(5):e0198142. doi: 10.1371/journal.pone.0198142 (PMC5967834; doi:10.1371/journal.pone.0198142)
Supplement: S4 Table — Λ and V represent upward and downward convexities in the plot of the bias of the most highly expressed miRNAs that meet the criterion of the Λ and V shapes, respectively. The upward convexity indicates that the miRNAs preferentially target genes with intermediate expression. Hyphens indicate a non-Λ-non-V-shaped plot or not available. Ante and Euth represent ante-eutherian and eutherian origins of miRNAs, respectively. C010, C020, and C030 are the mouse sets of predicted target sites by TargetScan Context++ Score in increasing order of stringency. Br, Brain; He, Heart; Ki, Kidney; Li, Liver; Ov, Ovary; Pl, Placenta; Te, Testis. (DOCX) [file pone.0198142.s013.docx]

| Set | Origin | Br | He | Ki | Li | Ov | Pl | Te | No. |
| --- | --- | --- | --- | --- | --- | --- | --- | --- | --- |
| C010 | Ante | - | V | - | - | Λ | - | - | 1 |
|  | Euth | - | - | - | Λ | - | - | - | 1 |
| C020 | Ante | - | - | Λ | Λ | Λ | - | - | 3 |
|  | Euth | - | - | - | - | - | - | - | 0 |
| C030 | Ante | - | V | - | - | Λ | - | Λ | 2 |
|  | Euth | - | - | - | V | - | - | - | 0 |
