# Supplementary material for: Human microRNAs preferentially target genes with intermediate levels of expression and its formation by mammalian evolution
Source: PLoS One. 2018 May 24;13(5):e0198142. doi: 10.1371/journal.pone.0198142 (PMC5967834; doi:10.1371/journal.pone.0198142)
Supplement: S5 Table — Ante and Euth represent ante-eutherian and eutherian origins of miRNAs, respectively. Hyphens indicate not available or “not in order”. C010, C020, and C030 are the mouse sets of predicted target sites by TargetScan Context++ Score in increasing order of stringency. *Derived from Wilcoxon signed-rank test (two-sided; see Materials and Methods) attesting different A between Ante and Euth over the mouse C0X0 matrix series as a whole. Br, Brain; He, Heart; Ki, Kidney; Li, Liver; Ov, Ovary; Pl, Placenta; Te, Testis. (DOCX) [file pone.0198142.s014.docx]

| Set | Origin | Br | He | Ki | Li | Ov | Pl | Te | p* |
| --- | --- | --- | --- | --- | --- | --- | --- | --- | --- |
| C010 | Ante | 27.6 | - | 2.1 | 5.3 | 2.5 | - | 2.7 | 0.0009 |
|  | Euth | - | - | - | - | - | - | - |  |
| C020 | Ante | 35.3 | - | 2.6 | 4.6 | 1.5 | - | 5.5 |  |
|  | Euth | - | 0.91 | 0.13 | - | - | - | - |  |
| C030 | Ante | 34.6 | - | 2.9 | 3.6 | 1.1 | - | 12.0 |  |
|  | Euth | - | 0.12 | 0.34 | - | - | 0.41 | - |  |
