# Supplementary material for: Human microRNAs preferentially target genes with intermediate levels of expression and its formation by mammalian evolution
Source: PLoS One. 2018 May 24;13(5):e0198142. doi: 10.1371/journal.pone.0198142 (PMC5967834; doi:10.1371/journal.pone.0198142)
Supplement: S6 Table — Ante and Euth represent ante-eutherian and eutherian origins of miRNAs, respectively. Hyphens indicate not available or “not in order”. C010, C020, and C030 are the mouse sets of predicted target sites by TargetScan Context++ Score in increasing order of stringency; P010, P020, and P030 are those predicted by PITA, so that each of them has the nearest number of target sites to that of mouse C0X0 sets. Br, Brain; He, Heart; Ki, Kidney; Li, Liver; Ov, Ovary; Pl, Placenta; Te, Testis. *Derived from Wilcoxon signed-rank test (two-sided; see Materials and Methods) attesting different A between Ante and Euth over the mouse C0X0 matrix series as a whole. (DOCX) [file pone.0198142.s015.docx]

| Set | Origin | Br | He | Ki | Li | Ov | Pl | Te | p* |
| --- | --- | --- | --- | --- | --- | --- | --- | --- | --- |
| C010 | Ante | - | - | - | - | 0.89 | - | 0.70 | 0.09 |
|  | Euth | - | 0.11 | - | - | - | - | - |  |
| C020 | Ante | - | - | 0.22 | - | 0.86 | - | 0.22 |  |
|  | Euth | - | 0.28 | - | - | - | - | - |  |
| C030 | Ante | - | - | - | - | 0.40 | - | 0.96 |  |
|  | Euth | - | 0.13 | 0.37 | - | - | - | - |  |
