# Supplementary material for: Human microRNAs preferentially target genes with intermediate levels of expression and its formation by mammalian evolution
Source: PLoS One. 2018 May 24;13(5):e0198142. doi: 10.1371/journal.pone.0198142 (PMC5967834; doi:10.1371/journal.pone.0198142)
Supplement: S8 Table — Ante and Euth represent ante-eutherian and eutherian origins of miRNAs, respectively. Hyphens indicate not available or “not in order”. C010, C020, and C030 are the sets of predicted target sites by TargetScan Context++ Score in increasing order of stringency. *Derived from Wilcoxon signed-rank test (two-sided; see Materials and Methods) attesting different A between Ante and Euth over each series of C0X0, P0X0 and both† matrices as a whole. Br, Brain; Ki, Kidney; Li, Liver; Pa, Pancreas; Th, Thyroid; Te, Testis. (DOCX) [file pone.0198142.s017.docx]

| Set | Origin | Br | Ki | Li | Pa | Th | Te | p* | p^†^ |
| --- | --- | --- | --- | --- | --- | --- | --- | --- | --- |
| C010 | Ante | 9.5 | 6.1 | - | 7.8 | 11.9 | - | 0.03 | 0.02 |
|  | Euth | - | 1.9 | 5.7 | - | 16.6 | - |  |  |
| C020 | Ante | 8.5 | 1.5 | 3.7 | 11.7 | 7.6 | - |  |  |
|  | Euth | - | - | 4.6 | - | - | - |  |  |
| C030 | Ante | 6.1 | 0.2 | 3.2 | 8.4 | 1.7 | - |  |  |
|  | Euth | - | - | 6.2 | - | - | - |  |  |
| P010 | Ante | 4.7 | 1.2 | 4.2 | 0.7 | 2.9 | 0.1 | 0.17 |  |
|  | Euth | - | - | 1.9 | - | 15.7 | - |  |  |
| P020 | Ante | - | 2.0 | 6.2 | - | 1.7 | 1.6 |  |  |
|  | Euth | - | - | - | - | 7.1 | - |  |  |
| P030 | Ante | - | 0.4 | 5.8 | 1.4 | 3.6 | 3.1 |  |  |
|  | Euth | - | - | 2.1 | - | 5.2 | 1.6 |  |  |
