# Supplementary material for: Human microRNAs preferentially target genes with intermediate levels of expression and its formation by mammalian evolution
Source: PLoS One. 2018 May 24;13(5):e0198142. doi: 10.1371/journal.pone.0198142 (PMC5967834; doi:10.1371/journal.pone.0198142)
Supplement: S1 Text — (DOCX) [file pone.0198142.s019.docx]

**S1 Text. Supporting Methods**

**Mouse 3′ UTR sequences**

Sequences and annotations of the mouse protein-coding transcripts registered in Ensembl 89 were downloaded through BioMart (http://www.ensembl.org/). The transcripts and genes assigned to chromosomes 1–19, X and Y were retained. In the same way as done for the human 3′ UTRs, when multiple transcripts were annotated for a gene, a representative transcript was chosen for each gene using the following criteria in decreasing order of priority: i) longer CDS (coding sequence), ii) a coding end located further downstream, iii) longer 3′ UTR, and iv) longer transcript. The 3′ UTR sequences of 8 bp or longer were obtained (n = 20,723).

**Acquisition of mouse miRNA information**

The annotations and sequences of mouse 1913 mature miRNAs linked to the 1193 precursor miRNAs by miRBase release-21 record descriptions were extracted, from which a 1913 × 1193 matrix ***m2h^(m)^*** was made in the same way as done for human.

**Mouse miRNA target prediction by TargetScan**

In the same way as for human, TargetScan 7.01, TargetScan Context++ Score (CS) and overlap removal processes were performed on mouse 20,723 3′ UTR sequences. The thresholds of CS < −0.4, −0.5 and −0.6 yielded predicted target sites of similar densities to those of human, 0.07 (human: 0.07), 0.044 (0.046), 0.03 (0.03) per mature miRNA per gene, which were named C010^(m)^, C020^(m)^ and C030^(m)^ sets, or generically ***g2m^(m)^*** matrix (20723 × 1913). Then, 20723 × 1193 matrix ***G2H^(m)^*** connecting a precursor to its target genes is computed as ***1****_A_*(***g2m^(m)^***·***m2h^(m)^***), in the same way as done for the human matrices.

**Expression intensity of mouse miRNAs**

Mouse miRNA precursor profile by Landgraf et al., i.e. their “Table S10” (mmc11.xls), was downloaded from ars.els-cdn.com/content/image/1-s2.0-S0092867407006046-mmc11.xls. Each of 1913 mouse precursors, whose name was identical to that of any of the human 1426 precursors, was selected as an ortholog; which amounted to a total of 350 mouse precursors. Of the 350, 229 precursors were able to be assigned the time of origin based on that of human; 135 to Ante-Eutherian, 92 Eutherian, two to Simian and 0 to Hominoid origins. Out of the 229, 149 mouse precursors had any expression signals in the Landgraf’s data set; 102 Ante-Eutherian and 47 Eutherian origins.

Clone counts were available for the samples of seven organs that were shared with the data set of protein-coding genes by De Rie *et al*. (described below); the seven organs were brain, heart, kidney, liver, ovary, placenta and testis, in which the clone counts of the mouse frontal cortex and midbrain were averaged as that of brain. In the same way as done for human, for each miRNA locus that had both the 3p- and the 5p-arms’ clones counts, the larger count was used; and the by-organ precursor expression intensity was represented by a 1913 × 7 matrix ***s2h^(m)^***.

**Expression intensity of mouse protein-coding genes**

De Rie’s data set “E-MTAB-3578-query-results.tpms.tsv” [20] was downloaded from EMBL-EBI Expression Atlas (https://www.ebi.ac.uk/gxa). In the data set, seven normal by-organ samples were comparable to those of the miRNA data set. For brain, ovary, pancreas, prostate, placenta and testis, adult normal mouse samples were used; although for organs lacking adult samples, i.e. heart, kidney and liver, juvenile samples were instead used. For mouse brain, the expression intensity of cerebral cortex, medulla oblongata and hippocampal formation were average. De Rie’s data set consisted of 18835 genes, of which the Ensembl (release 89) gene IDs were matched for 16390 genes within the 20723 genes of the 3′ UTR set. The clone counts of 16390 genes, which is provided by TPM (transcripts per kilobase million), were used for mouse gene expression intensities. Then, the expression intensity of a gene (*g*) for each organ (*z*), ***s2g^(m)^_[g,z]_***, where *g* stands for a gene *g* ∈ {1, 2, ..., 20723}, and *z* for an organ *z* ∈ {1, 2, ..., 7}.

**Microarray-based expression intensity of human miRNAs**

A data set of microarray-based human miRNA expression intensity by Ludwig N et al. [21], data_matrix_raw.txt, was downloaded from ccb-web.cs.uni-saarland.de/tissueatlas/. The signal intensities included minus values due to background subtraction of their internal procedure; the minus values were treated as 0 expressions following their description in the paper. In this data set, seven organs corresponded to those of the protein-coding genes’ expression data set by Uhlén et al. [16], i.e. brain, kidney, liver, pancreas, prostate, thyroid and testis. The clone counts for kidney, liver, thyroid and testis, which each had two samples, were averaged.

The Ludwig’s data set represented expression intensities of mature miRNAs. From the values of mature miRNAs, a matrix, ***s2m^(L)^_[μ,z]_*** was made, where *μ* stands for a mature miRNA *μ* ∈ {1, 2, ..., 1935}, and *z* for an organ *z* ∈ {1, 2, ..., 7}. Each value of ***s2m^(L)^,*** to which NA (not available) was assigned, was changed to 0 to operate the following matrix multiplication. As a converting matrix, ***M2H*** was given as *M2H_[μ,h]_* = *m2h_[μ,h]_/*Σ*_μ_(m2h_[·,h]_)*, so that each precursor, *h* ∈ {1, 2, ..., 1426}, corresponds to a total of one mature miRNA even in the cases where a precursor has multiple mature miRNAs. Then, the expression intensity of a precursor (*h*) for each organ (*z*), ***s2h^(L)^_[h,z]_***, was computed as (***M2H****)****^T^·s2m^(L)^***.
